# Detecting Burnout Among Undergraduate Computing Students with Supervised Machine Learning

**DOI:** 10.3390/healthcare13233182

**Published:** 2025-12-04

**Authors:** Eldar Yeskuatov, Lee Kien Foo, Sook-Ling Chua

**Affiliations:** Faculty of Computing and Informatics, Multimedia University, Persiaran Multimedia, Cyberjaya 63100, Malaysiaslchua@mmu.edu.my (S.-L.C.)

**Keywords:** academic burnout, machine learning, burnout detection, well-being, student mental health

## Abstract

**Background:** Academic burnout significantly impacts students’ cognitive and psychological well-being and may result in adverse behavioral changes. An effective and timely detection of burnout in the student population is crucial as it enables educational institutions to mobilize necessary support systems and implement intervention strategies. However, current survey-based detection methods are susceptible to response biases and administrative overhead. This study investigated the feasibility of detecting academic burnout symptoms using machine learning trained exclusively on university records, eliminating reliance on psychological surveys. **Methods:** We developed models to detect three burnout dimensions—exhaustion, cynicism, and low professional efficacy. Five machine learning algorithms (i.e., logistic regression, support vector machine, naive Bayes, decision tree, and extreme gradient boosting) were trained using features engineered from administrative data. **Results:** Results demonstrated considerable variability across burnout dimensions. Models achieved the highest performance for exhaustion detection, with logistic regression obtaining an F1 score of 68.4%. Cynicism detection showed moderate performance, while professional efficacy detection has the lowest performance. **Conclusions:** Our findings showed that automated detection using passively collected university records is feasible for identifying signs of exhaustion and cynicism. The modest performance highlights the challenges of capturing psychological constructs through administrative data alone, providing a foundation for future research in unobtrusive student burnout detection.

## 1. Introduction

Academic life can be a stressful and challenging experience. University students often face high cognitive demands, financial difficulties, pressure to perform, stress associated with examinations and deadlines, and societal and family expectations [1,2,3]. These challenges of academic life, compounded by the lack of social support and ineffective coping mechanisms, place students at risk of burnout.

Within the academic context, burnout was defined by ref. [4] as “feeling exhausted because of study demands, having a cynical and detached attitude toward one’s study, and feeling incompetent as a student.” This definition of academic burnout is analogous to occupational burnout, mirroring Maslach’s three-dimensional occupational burnout model, which consists of exhaustion, cynicism, and professional efficacy [5]. However, these elements of burnout manifest distinctly in the educational setting. Particularly, the exhaustion dimension encompasses both physical and emotional fatigue resulting from studies in general, as well as specific activities, such as attending classes. The cynicism dimension represents feelings of indifference and detachment, frequently manifested as loss of interest in studies, and doubts about the significance or contribution of one’s work. Professional efficacy reflects a student’s perceived academic competence, specifically the satisfaction with accomplishments and effectiveness in the studies.

Given students’ susceptibility to burnout, researchers have investigated its prevalence in the academic setting. Although burnout has been examined in various countries and cultural contexts, most studies have focused on medical students. These studies demonstrate considerable variation in reported student burnout rates. For instance, in a systematic review of studies on medical student burnout, ref. [6] uncovered that the prevalence rates ranged from 45% to 71%. Similarly, ref. [7] reported a broader range of burnout rates from 7% to 75%.

In addition to distinct characteristics of academic environments, the substantial variability in reported rates stems from the use of different instruments for measuring burnout. These instruments include the Copenhagen Burnout Inventory, Maslach Burnout Inventory (MBI), Oldenburg Burnout Inventory, and Work-Related Behavior and Experience Patterns Scale. Generally, the MBI emerges as the most prevalent instrument utilized in burnout research. Nonetheless, the inconsistency of prevalence rates can also be observed among studies employing the same assessment tools. In this case, the inconsistency is partly attributed to the use of different cutoff criteria for burnout symptomology and varying operational definitions.

Research on academic burnout has revealed that it significantly impacts multiple aspects of a student’s life. These consequences can be broadly categorized into cognitive, psychological, physiological, and behavioral effects. The cognitive effects of burnout can harm performance, impair decision-making, and lead to increased errors [8,9]. Burnout can also result in depression, anxiety, and eating disorders, with more serious cases resulting in suicidal ideations [1,2,10]. Physiological impacts can manifest as chronic fatigue, migraines, and disrupted sleep patterns [11]. Also, academic burnout has been associated with adverse behavioral changes: higher rates of absenteeism and dropout, diminished motivation and engagement, academic misconduct, and, in severe instances, substance use, aggression, and social isolation [12,13,14,15,16].

Considering the substantial consequences of burnout, it is imperative to effectively detect and measure burnout in the student population. Timely identification potentially enables educational institutions to mobilize necessary support systems and implement appropriate intervention strategies. Currently, burnout is primarily detected through the administration of inventories [17]. The typical assessment process involves individuals completing online or paper-based questionnaires where responses are measured on a Likert scale, e.g., ranging from “never” to “every day”. This method of assessment is widely utilized and well-established in burnout research. However, there are several limitations associated with the administration of psychological surveys.

The foremost limitation is the administrative overhead related to the distribution of surveys, collection of responses, and subsequent analysis of results. These administrative demands not only require significant research resources but could also result in delays that may compromise the reliability of findings, specifically in longitudinal studies where timing is critical. In addition to procedural challenges, psychological surveys are susceptible to response biases that may distort findings. For instance, the self-reported psychological measures can be influenced by the mood of participants at the time of the survey [18]. These survey results may inaccurately reflect the general emotional state of study subjects. Psychological inventories can also be impacted by the social desirability bias. It refers to the tendency of respondents to underreport their negative traits and behaviors, and to overreport the more socially desirable ones, presenting a skewed positive self-image [19]. Such positively biased self-reporting can occur in anonymous surveys as well [20] and can happen unconsciously [21]. Another pervasive type of bias is the extreme response bias, which occurs when participants consistently choose the highest or the lowest responses on the scale [22]. Moreover, the defensiveness of participants—characterized as denial or minimization of symptoms—can invalidate test results [23].

Machine learning emerges as an alternative to traditional burnout assessment. Machine learning algorithms work by analyzing data to detect patterns indicating the presence of burnout. These algorithms require input data, called features, which are quantifiable measurements that capture relevant information about individuals. Such applications can process large datasets, capturing patterns and interactions between multiple predictive factors. Given these potential advantages, several studies attempted to detect burnout using machine learning.

However, a significant limitation of these studies lies in their continued reliance on survey-based data for feature generation. For instance, ref. [24] utilized responses from multiple questionnaires as input features, including the Problematic Internet Use Questionnaire, Beck Depression Inventory, Athens Insomnia Scale, and Quality of Life Questionnaire. By using manually collected survey responses for feature engineering, these machine learning approaches constrain their practicality for automated detection and inherit the same limitations as traditional survey-based methods.

Another major limitation in this domain of burnout research is that it primarily focuses on healthcare workers, particularly physicians and nurses. While some studies examined student burnout, they primarily targeted medical and dental students [25,26,27]. Due to this gap, the non-medical student population remains underrepresented in burnout detection research.

The methodological limitations of existing burnout detection approaches, combined with an overwhelming focus on healthcare populations, present an important research opportunity. To address these challenges, we propose a machine learning-based student burnout detection model that does not rely on psychological survey responses to engineer features. Our approach leverages existing student records as potential indicators of academic burnout, eliminating the need for direct data collection from study participants. Consequently, it could mitigate the limitations associated with survey-based methods by reducing the administrative burden of surveys and avoiding response bias issues. To the best of our knowledge, this is the first approach that relies solely on university records to detect burnout among students.

The remainder of this paper is organized as follows. Section 2 presents a review of related work in academic burnout detection. Section 3 describes the methodology, including data collection, feature engineering, model training, and evaluation. Section 4 presents the results. Section 5 discusses the implications of findings, while Section 6 concludes the paper.

## 2. Related Work

### 2.1. Survey Data

Several studies have employed machine learning to detect burnout from different sources, with most utilizing survey responses as the primary data source. Specifically, researchers used various inventories to extract features potentially capturing burnout-signifying factors.

Ref. [28] surveyed 240 caregivers using the Caregiver Reaction Assessment Scale, World Health Organization Quality of Life, and Hospital Anxiety and Depression Scale inventories. These inventories were used to assess caregivers’ overall psychological well-being. The survey responses were then combined with other caregiver and patient characteristics (e.g., age, gender, and medical history) as features. These features were used to train various machine learning algorithms, such as decision tree (DT), random forest (RF), and gradient-boosted decision trees (GBDT). Using 3-fold cross validation, the findings from this study showed that machine learning is effective in detecting caregiver burnout, with GBDT achieving an F1 score of 86%.

Ref. [29] took a different approach by designing a custom survey to collect demographic characteristics and assess work system factors. The survey was administered to 450 healthcare professionals. Demographic characteristics included job position, gender, race, and marital status, whereas work factors represented their job demands and resources, such as time pressure and lack of research support. These survey-based features were used to train an RF model to detect the presence of burnout. To avoid overfitting, 5-fold cross-validation was conducted. Their results showed that RF was effective in detecting burnout among healthcare professionals, achieving an area under the curve (AUC) score of 0.81.

Similarly, ref. [30] assessed the workplace environment of 4029 nurses by administering the Guarding Minds at Work questionnaire. The resulting 13 workplace features were used to train an RF model, which was evaluated using 10-fold cross-validation. However, this study approached burnout detection as a regression task, predicting three burnout component scores: emotional exhaustion, depersonalization, and personal accomplishment.

While most studies focused on healthcare workers, ref. [31] surveyed 303 university students using the Inventory of Socially Supportive Behaviors and a custom self-regulated learning questionnaire. The survey responses were used to create features representing students’ learning patterns and received social support. Several models were trained with resulting features to detect the presence of burnout and evaluated with 10-fold cross-validation, with the Bayesian Network model obtaining the highest F1 score of 70%.

Ref. [32] survey 274 psychology students using MBI and questionnaires assessing sociodemographic and clinical characteristics, psychological distress, psychological well-being, difficulties in emotional regulation, sleep quality, physical activity, and diet. Based on these survey responses, the authors clustered students into burnout and non-burnout profiles. The resulting profiles were then used as the target labels, which were predicted using features derived from the survey responses. To prevent overfitting and ensure generalizability, the study implemented 10-fold cross-validation. Their best-performing model, extreme gradient boosting (XGBoost), achieved an F1 score of 94.73%.

The reliance of these studies on surveys for feature engineering limits their practicality for automatic burnout detection and introduces additional complexity to their methodology. To address this issue, several studies attempted to detect burnout from other sources.

### 2.2. Social Media Data

Among these alternative approaches, social media platforms emerged as a potential medium for detecting burnout. The main advantage of this data source is that it provides unobtrusive access to readily available online data, eliminating the need for survey administration.

Ref. [18] analyzed Reddit posts to detect burnout using natural language processing techniques. The authors extracted bag-of-words features from 13,568 Reddit posts and evaluated multiple classification algorithms using 10-fold cross-validation: logistic regression (LR), RF, support vector machine (SVM), and their proposed ensemble model. Their best-performing ensemble model achieved 93% accuracy and an F1 score of 43% on an unbalanced test dataset.

Similarly, ref. [33] aimed to detect burnout among Weibo social media users. Unlike the previous study, in addition to textual data, they analyzed post metadata—such as timestamp, number of likes, comments, and reposts—to generate features from 142,859 posts. LR, SVM, DT, RF, and XGBoost were trained and evaluated. However, the evaluation protocol is not specified in this study. The results demonstrated that XGBoost outperformed the other models in detecting burnout in Weibo users, achieving an F1 score of 78.13%.

Although social media can be a promising avenue for burnout detection, it is essential to consider that this approach requires the target population (e.g., physicians, nurses, or university students) to have an active social media presence. This reliance on social media activity omits individuals with limited or no online presence, potentially limiting the generalizability of this approach.

### 2.3. Biometric Data

In addition to behavioral indicators, researchers have explored objective biometric measurements—such as heart rate and sleep patterns—as potential sources for burnout detection. This approach is grounded in established findings that burnout has physiological impacts. These studies attempted to build models that capture physiological changes to detect burnout. However, the research on burnout detection through biometric data is still limited, with only a few studies exploring this approach.

Ref. [34] collected biometric data, including steps, heart rate, and sleep patterns, using wearable devices (Fitbit Charge 3) from 75 healthcare professionals working shifts at intensive care units and emergency rooms over five weeks. However, this study also manually collected survey responses to measure caffeine and alcohol consumption, naps, and overtime hours, not exclusively relying on automatic sensor measurements. The resulting features were used to train SVM, LR, and RF, which were evaluated using 5-fold cross-validation. SVM achieved an F1 score of 99% and an AUC score of 0.99. The findings from this study demonstrated that the sleep pattern and heart rate features were important predictors of burnout in healthcare workers.

The predictive capacity of heart rate was also investigated by ref. [35]. In this study, the ECG measurements were collected from 1615 healthcare workers using a medical-grade electrocardiograph to create 12 heart rate variability features. The ECG features were combined with sociodemographic and medical history features to train six models, including RF, CatBoost, Extra-Trees, XGBoost, *k*-nearest neighbors (KNN), and GBDT. A single train-test split was used in this study, although the ratio was not specified. The Extra-Trees classifier achieved the highest performance, obtaining an F1 score of 80% and an AUC of 0.84. This study discovered that heart rate was an informative feature for detecting burnout.

While these studies obtained high classification performance, it is crucial to note that their approaches also incorporated manually collected survey-based features, which limit the applicability for automatic real-time burnout detection. In addition to that, using raw biometric data for feature engineering requires substantial domain knowledge in medicine. Despite these limitations, the existing studies were able to demonstrate that biometric features could be valuable for burnout detection.

### 2.4. Electronic Health Records

The research in this domain has also investigated the potential of electronic health records (EHR) to identify patterns indicative of burnout. One of the advantages of this approach is that it does not require active data collection. Instead, EHR systems routinely generate audit logs documenting various clinical activities—such as patient interactions, laboratory tests, and report updates—allowing unobtrusive tracking of the workload.

Ref. [36] aimed to develop a burnout detection tool using passively collected records. The EHR audit logs were collected for 88 trainee physicians over six months to engineer workload (e.g., number of patients per day) and temporal (e.g., time gaps between consecutive activities) features. This study approached burnout detection as both regression and classification tasks, using the resulting features to train linear regression, LR, SVM, multilayer perceptron (MLP), RF, and GBDT. The models were evaluated using 10-fold cross-validation. However, their results demonstrated that EHR-based features had poor discriminative ability, with the best-performing RF model achieving an AUC of 0.595.

Ref. [37] proposed an end-to-end hierarchical deep learning framework utilizing the EHR activity log dataset used in [36]. Their framework includes an activity embedding layer that learns temporal representations of physician activities, eliminating the need for manual feature engineering. The hierarchical nature of the framework allows the modeling of physician behavior on multiple temporal levels (e.g., hours, shifts, days, and months). Having applied 5-fold cross-validation, this study found that their proposed model achieved an AUC of 0.648, improving on the results from the previous study.

Ref. [17] similarly evaluated the ability of EHR-based features to predict both continuous burnout score and binary burnout status. The researchers utilized the EHR activity logs of 233 primary care physicians to engineer the workload and efficiency features. These features were used to train five models (GBDT, LR, RF, KNN, and MLP), which were evaluated using a single 80-20 train-test split. LR achieved the highest AUC score of 0.63 in predicting binary burnout status. The authors concluded that the EHR features had a limited ability to detect burnout among physicians.

In general, the research using EHR features for burnout detection is still in its early stages, with few studies conducted to date. Moreover, this approach is only applicable to healthcare personnel, with best-performing models achieving modest AUC scores between 0.60 and 0.65.

## 3. Materials and Methods

### 3.1. Study Participants

For this cross-sectional study, we recruited 688 students enrolled in Information Technology (IT) and Computer Science (CS) programs at a Malaysian private university. Participating students were pursuing either foundation, diploma, or bachelor’s degrees. This study was conducted between October 2023 and June 2024. A convenience sampling approach was used for participant recruitment. The survey was distributed to students enrolled in two faculties offering computing programs. Study participation was voluntary, with students receiving no financial incentives for their involvement.

### 3.2. Data Collection

To implement supervised machine learning for burnout detection, two essential components are required: the input features representing student characteristics (predictors), and the corresponding labels indicating students’ burnout status (target variable). The labeled dataset enables the model to learn the relationships between student characteristics and burnout outcomes.

#### 3.2.1. Target Variables

The primary outcome of this study is to predict the presence of burnout. In this study, we operationalized student burnout using Maslach’s three-dimensional model, which characterizes the syndrome using exhaustion (EX), cynicism (CY), and professional efficacy (PE). Exhaustion signifies feeling emotionally depleted by academic demands (e.g., feeling drained by examinations and coursework). Cynicism manifests as a detached, negative attitude toward academic work and studies (e.g., questioning the value of education). Professional efficacy represents a sense of competence and achievement in academic work (e.g., considering oneself a good student). The Maslach Burnout Inventory (MBI) consistently demonstrates sound psychometric properties and is commonly used as an assessment tool in burnout research. Therefore, we have utilized the MBI General Survey for Students (MBI-GS(S)) to evaluate participants’ burnout levels.

MBI-GS(S) comprises five statements measuring the EX dimension, five measuring the CY dimension, and six measuring the PE dimension. For each of these 16 statements, participants indicated how frequently they experienced the described feeling using a 7-point Likert scale, with scores ranging from 0 (never) to 6 (every day). The final score for each burnout dimension was calculated by averaging the scores of the statements corresponding to that dimension.

Although the MBI is an extensively validated tool, it does not provide definitive thresholds that signify burnout. Moreover, there is no consensus in the burnout research community on the specific cut-off criteria for low and high values of burnout dimensions, with studies employing various thresholds [38]. Therefore, we selected a threshold of three based on the conceptual meaning of the MBI frequency scale. A value of three corresponds to “a few times a month” and represents a distinction between occasional experiences (values 0–2: “never” to “once a month or less”) and recurring experiences (values 3–6: “a few times a month” to “every day”). This frequency-based distinction reflects an understanding that for a student to be considered burned-out, their symptoms need to be persistent rather than occasional. This threshold was also utilized to dichotomize MBI scores in similar studies on healthcare workers [29,39]. The frequency scale is consistent across all versions of the MBI, including the MBI-GS(S) and versions designed for healthcare workers and other occupational groups. Therefore, the interpretation of three (“a few times a month”) carries the same conceptual meaning across populations.

Using the MBI-GS(S), we collected three scores for each student: EX, CY, and PE. Subsequently, these continuous burnout scores were dichotomized using the threshold of three. Specifically, for EX and CY, scores equal to or greater than three (EX or CY ≥ 3) were coded as 1 (burnout), while scores less than three (EX or CY < 3) were coded as 0 (no burnout). However, for PE, scores equal to or less than three (PE ≤ 3) were coded as 1 (burnout), while scores more than three (PE > 3) were coded as 0 (no burnout). The PE coding was reversed, as the MBI conceptualizes burnout as high exhaustion, high cynicism, and low professional efficacy. Consequently, this coding transformed survey results into binary labels for each of the three burnout components. The resulting distribution of classes is shown in Table 1.

#### 3.2.2. Predictor Variables

The university records of the participating students were collected from the administration. To anonymize the data, the student names were excluded from the dataset. The university records served as the sole data source for our predictive features. The information in these records could be broadly categorized as demographic, academic, and institutional variables. Demographic characteristics include variables such as nationality, race, gender, and place of residence. Academic records include performance indicators such as cumulative grade point average (GPA) and completed credit hours. Institutional information encompasses variables such as current term, enrollment status, faculty, campus, and program. No additional data collection methods—such as surveys or external assessments—were employed to construct the input features. During the initial examination, we identified 123 entries with no academic records as they belonged to first-semester students. After excluding these cases, the final dataset consists of 565 student records.

### 3.3. Feature Engineering

#### 3.3.1. Data Cleaning

The original student records consisted of 32 variables, with some containing null values and duplicating information. Having conducted the exploratory data analysis, we identified and excluded variables that were irrelevant for burnout detection.

The variables *Program Action*, *Admit Term*, and *Plan Code* were removed from the dataset. *Program Action* is used by the administration to encode the current action of the program. *Admit Term* contains the trimester during which the student was admitted to the university. *Plan Code* contains the code assigned to the student’s study plan. These categorical variables contained a high number of unique values. For example, *Admit Term* had 18 distinct categories. Including these high-cardinality categorical variables would increase the dimensionality of the final feature set, potentially compromising model performance.

The original dataset contained six variables pertaining to home and mailing addresses. We excluded these variables for two primary reasons. These legally required records—often taken from national identification documents—may not reflect students’ actual places of residence, as many students reside on campus or in nearby areas. Moreover, these address variables contained numerous unique values that would substantially increase the feature dimensionality after encoding. For example, the *City Home* variable contained 119 unique cities.

Additionally, we removed the *Current GPA* variable as 34.7% (196/565) of values were missing. Given the high rate of missing values, we did not conduct data imputation to avoid introducing bias. Instead, we utilized *Cumulative GPA* as the indicator of academic performance, which had complete data for all participants.

#### 3.3.2. Feature Transformation

Given that most variables in the student records are categorical and contain numerous unique values, we applied several transformations to convert them into meaningful features.

*Current Term* indicates which trimester the student is completing. An academic year consists of one short and two long trimesters. Based on the university’s coding scheme, we transformed this variable into a binary feature indicating whether the student is completing a long or short trimester. Longer trimesters typically involve a higher workload as students take more subjects than they would in short trimesters. This transformed feature could capture the relationship between academic workload and burnout.

*Academic Level* contains codes (“alpha”, “beta”, “gamma”, and “delta”) that reflect a student’s academic year. However, the meaning of these codes varies by degree. For example, “beta” indicates second-year studies for diploma students, whereas for bachelor’s degree students it signifies first-year studies. Therefore, using degree information, we transformed this variable to create an ordinal feature that consistently represents the year of study across all programs.

The variables *Program Code*, *Program Description*, and *Program Short Description* were combined into a single feature *Program* as they contained identical information about the student’s program, only using different naming conventions (e.g., “DD16”, “Diploma in Information Technology”, “Dip. I.T.”). Similarly, *Faculty ID* and *Faculty Description* were combined to create a single *Faculty* feature. The consolidation of these features eliminated redundancy while preserving the relevant information.

The original *Nationality* variable contained 17 unique categories, with 10 categories containing only one example. Additionally, 93.6% (529/565) of the students were from Malaysia. Therefore, this variable was transformed into a binary feature indicating whether the student is local or international. This transformation is based on the premise that international and local students may encounter different academic and social challenges during their studies, which potentially influence their susceptibility to burnout.

The *Race* variable originally contained eight categories. To reduce cardinality, categories with very few examples (e.g., the “B. SARAWAK” category with two students) were combined with the existing “Others MYS” category. This consolidation maintains information about racial diversity while minimizing sparse categories that could hinder model performance.

The *Scholarship* variable contained only one data value. Therefore, we combined it with *Sponsorship* and named the combined variable *Financial Assistance*. However, there were two issues with *Financial Assistance*: 92% of values were null, and the remaining 8% were distributed across 11 unique categories. To address these issues, we converted it into a binary feature that denotes whether the student receives any form of financial assistance (i.e., yes/no). The variables *Discount*, *MUET Score*, and *Loan* exhibited similar issues: predominantly null values and high cardinality. Therefore, we transformed each into a corresponding binary feature indicating presence or absence of that attribute.

As a result of data cleaning and feature transformation, the final dataset consisted of 18 features. Before training the models, nominal categorical features were further processed using one-hot encoding. This transformation ensures that machine learning algorithms do not derive any inherent ordering among discrete categories. Table 2 presents the complete feature set with descriptions.

### 3.4. Model Training

In this study, we separated burnout detection into three subproblems addressing EX, CY, and PE classification. To detect burnout, the algorithms predict whether students fall into the burnout or no-burnout class for each dimension. Five algorithms were trained for each burnout subproblem: naïve Bayes (NB), logistic regression (LR), support vector machine (SVM), decision tree (DT), and extreme gradient boosting (XGBoost). These algorithms were selected to represent different classification approaches. In particular, DT represents a tree-based learner, XGBoost represents an ensemble of tree-based learners, LR and SVM are linear classifiers, and NB is a probabilistic classifier. NB, LR, SVM, and DT were implemented with the scikit-learn library (version 1.7.2), while XGBoost (version 1.7.4) was implemented using its scikit-learn interface. Table 3 lists the key hyperparameters and their default values used in each model. Given the dataset size (*n* = 565), we did not train neural network models, which typically require several thousand samples for optimal performance.

#### 3.4.1. Naïve Bayes

The naïve Bayes algorithm applies Bayes’ theorem to calculate the probability of the target outcome given the input features [40]. For burnout prediction, given a new student’s characteristics (e.g., *Program*, *Gender*, and *Cumulative GPA*), NB calculates two probabilities: the probability of a student experiencing burnout P(B|C), and the probability of not experiencing burnout $P(\bar{B}|C)$. Here, B and $\bar{B}$ represent the student belonging to the burnout and non-burnout classes, respectively, while C represents the set of characteristics. The algorithm outputs the class with the highest probability as the final prediction.

#### 3.4.2. Logistic Regression

Logistic regression models the relationship between the student characteristics and the target burnout class. Despite its name, LR is a classification algorithm that produces discrete class predictions: burnout or no burnout. The relationship can be expressed as Y = σ(Xw+b), where X is the set of student characteristics, w is the set of weights assigned to each student characteristic, and b represents the bias term [41]. During training, the algorithm learns the values for w and b, assigning higher weights to characteristics more strongly associated with the target class.

To predict burnout in a new student, the algorithm passes the weighted summation of characteristics $\left(X_{new}w+b\right)$ through a sigmoid function σ to determine the probability of the student experiencing burnout $Y_{new}$. Based on the calculated probability, LR assigns students to discrete classes using a 0.5 threshold: probability ≥ 0.5 indicates burnout, whereas probability < 0.5 indicate no burnout.

#### 3.4.3. Support Vector Machine

Support vector machine is a classification algorithm that uses a decision boundary, called a hyperplane, to separate students into burnout and non-burnout classes in the feature space, where each student is represented as a data point based on their characteristics. During the training phase, SVM determines the optimal hyperplane with the largest margin (i.e., the largest distance to the nearest data points in each class), where the data points closest to the hyperplane are also called support vectors, as they define where the hyperplane should be placed [42].

A large margin allows SVM to be flexible and to generalize well to unseen data. For prediction, new students are classified based on which side of the hyperplane they fall on. Those on one side are classified as experiencing burnout, while those on the other side are classified as not experiencing burnout.

#### 3.4.4. Decision Tree

Decision tree is a rule-based classification algorithm that uses a tree-like structure of decision paths to predict the target class [43]. During training, DT builds an upside-down tree with the root at the top. Starting at the root node, the algorithm first selects a characteristic that best splits the dataset into burnout and non-burnout classes. For example, one way is to divide students based on their campus, which creates two branches with different subgroups of students. At the new decision nodes for each subgroup, DT then selects another characteristic to further split students: for example, if *Cumulative GPA* ≤ 2.0. The partitioning is repeated at each new node until the branches are “maximally pure” (containing only burnout or only non-burnout cases) or until the tree reaches the maximum size limit [44].

As a result, the algorithm creates a hierarchy of if-else rules that lead to the target class [45], making DT highly interpretable and intuitive. To predict the burnout status of a new student, the algorithm starts at the root and descends through the tree, choosing branches based on the student’s characteristics, until it reaches a terminal node (leaf) that corresponds to the final predicted class.

#### 3.4.5. Extreme Gradient Boosting

The predictive performance can be improved by combining several decision trees into an ensemble, for example, by applying a boosting technique. Boosting builds models sequentially, where each successive model is trained using the information from the previous one [46]. XGBoost is one of the commonly used machine learning algorithms that implements the boosting technique [47].

XGBoost begins by making a simple prediction (e.g., average likelihood of burnout from the training dataset) and calculates the error resulting from this initial prediction. The algorithm then trains a new tree to predict this initial error and adds the new tree to the overall model. Following that, the updated model makes a new prediction, and errors are calculated again. The process repeats for a predefined number of iterations or until the model’s error falls to an acceptable level. As a result of this iterative refinement, XGBoost creates a chain of decision trees, where the final model represents the cumulative knowledge of all trees.

### 3.5. Model Evaluation

In this study, we employed a stratified 5-fold cross-validation, where 80% of the data was used for training and 20% for testing. Classification performance was assessed with accuracy, precision, recall, and F1 score. The average scores across five folds are reported as final results.

**Figure 1:**
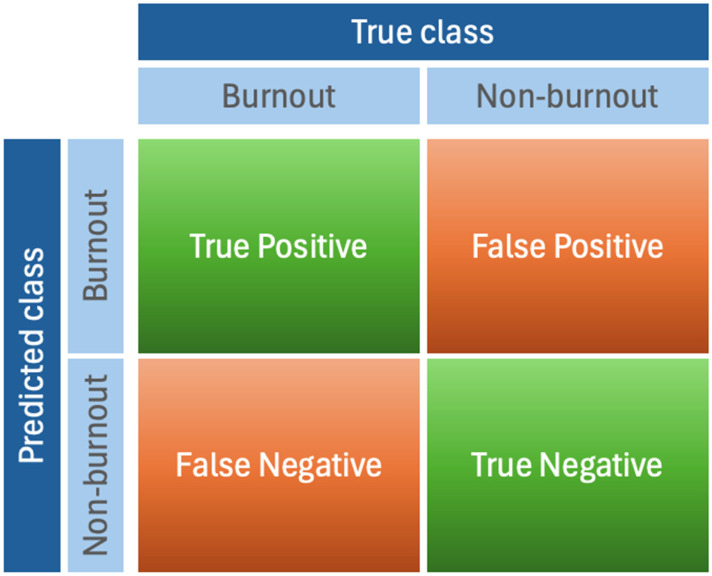
Confusion matrix for burnout detection.

This set of metrics is particularly important in burnout prediction as it is crucial to both correctly identify burned-out students (minimizing missed cases) and to avoid false alarms (preventing unnecessary interventions). These metrics are derived from a confusion matrix, which compares predicted classes with actual labels [48]. In the case of burnout prediction, as shown in Figure 1, the confusion matrix consists of: True Positives (*TP*)—correctly classified burnout cases; True Negatives (*TN*)—correctly classified non-burnout cases; False Positives (*FP*)—non-burnout cases incorrectly classified as burnout; and False Negatives (*FN*)—burnout cases incorrectly classified as non-burnout.

*Accuracy* measures the overall effectiveness of a model and is defined as the ratio of correctly identified examples:

(1) $$Accuracy\;=\;\frac{Number\;of\;correct\;predictions}{Total\;number\;of\;predictions}\;=\;\frac{TP+TN}{TP+TN+FP+FN}$$

While accuracy is an intuitive measure, it can be misleading when the testing set is imbalanced [44]. For example, if only 10% of students experience burnout, the model can achieve a 90% accuracy by always predicting no burnout on all test samples.

*Precision* measures how many of the students, predicted by the model as burned-out, actually have burnout:

(2) $$Precision\;=\;\frac{Correctly\;predicted\;burnout\;cases}{All\;cases\;predicted\;as\;burnout}\;=\;\frac{TP}{TP+FP}$$

Higher precision signifies fewer false positive predictions. Therefore, it may be prioritized in applications, where there is a higher cost of false alarms [45]. For example, in spam detection, it may be more tolerable for some spam emails to pass through than to miss an important email that got incorrectly labeled as spam.

*Recall* measures how many actual burnout cases were correctly predicted:

(3) $$Recall\;=\;\frac{Correctly\;predicted\;burnout\;cases}{All\;actual\;burnout\;cases}\;=\;\frac{TP}{TP+FN}$$

Higher recall means fewer false negative predictions, i.e., fewer missed burnout cases. Recall may be emphasized in domains where it is critical to minimize the number of missed cases, e.g., cancer diagnosis [49].

There is generally a trade-off between precision and recall, as improving one may worsen the other [50]. In addition, these metrics can be affected by the class imbalance, and, depending on the application, it may not always be apparent which metric to optimize for. Therefore, to assess a model that effectively balances these two competing metrics, the *F*1 score is utilized. *F*1 score is the harmonic mean of precision and recall:

(4) $$F1\;=\;2\times \frac{Precision\times Recall}{Precision+Recall}$$

For burnout prediction, balancing of precision and recall is crucial. While missing at-risk students potentially delays necessary support, false alarms could exhaust limited counseling resources. Therefore, a balanced approach is often practical for educational institutions.

## 4. Results

### 4.1. Exhaustion Detection Performance

**Figure 2:**
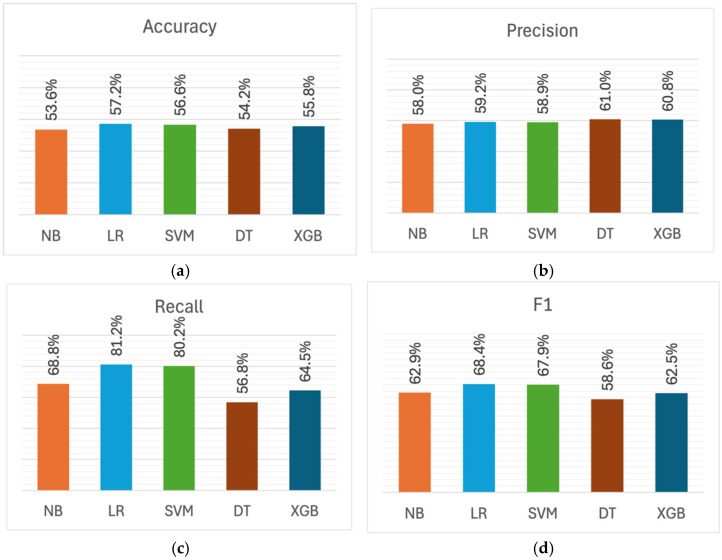
Performance metrics for exhaustion detection across machine learning models: (**a**) Accuracy; (**b**) Precision; (**c**) Recall; (**d**) F1 score.

Model evaluation was structured around three experiments, each predicting a class under a separate burnout dimension. Figure 2 presents the performance metrics achieved by models in detecting exhaustion. As shown in Figure 2a, the accuracy scores indicate comparable performance across models, ranging from 53.6% to 57.2%. LR achieved the highest accuracy at 57.2%, while NB recorded the lowest at 53.6%. A similar observation can be made about precision in Figure 2b: all models achieved comparable precision, with values ranging between 58% (for NB) and 61% (for DT).

In contrast, the differences between models become apparent upon analysis of recall and F1 metrics. As can be seen from Figure 2c, LR and SVM recorded the highest recall scores of 81.2% and 80.2%, respectively, substantially outperforming the other models by 12–24 percentage points. In comparison, DT achieved the lowest recall, recording 56.8%. A similar trend can be seen in F1 scores shown in Figure 2d: LR and SVM demonstrated the highest performance, obtaining 68.4% and 67.9%, respectively, while DT recorded the lowest F1 score of 58.6%.

Most models, except DT, tend to have higher recall than precision. This pattern is particularly evident in LR and SVM: LR showed the largest precision-recall gap at 22 percentage points (59.2% vs. 81.2%), followed by SVM at 21.3 percentage points (58.9% vs. 80.2%). The tendency for higher recall than precision indicates that these models produced a higher number of false positive predictions. In the context of burnout detection, this signifies that the models tend to err on the side of caution by over-identifying exhaustion cases. Specifically, they are more likely to flag students as exhausted when they are not, rather than missing students who are indeed exhausted. On the other hand, DT obtained higher precision (61%) relative to recall (56.8%), although with a smaller gap of approximately 4 percentage points.

Overall, LR and SVM demonstrated superior performance in detecting exhaustion compared to other models. Both achieved the best performance in three out of four metrics (accuracy, recall, and F1), while DT achieved the highest precision, although only outperforming LR and SVM by approximately two percentage points.

### 4.2. Cynicism Detection Performance

**Figure 3:**
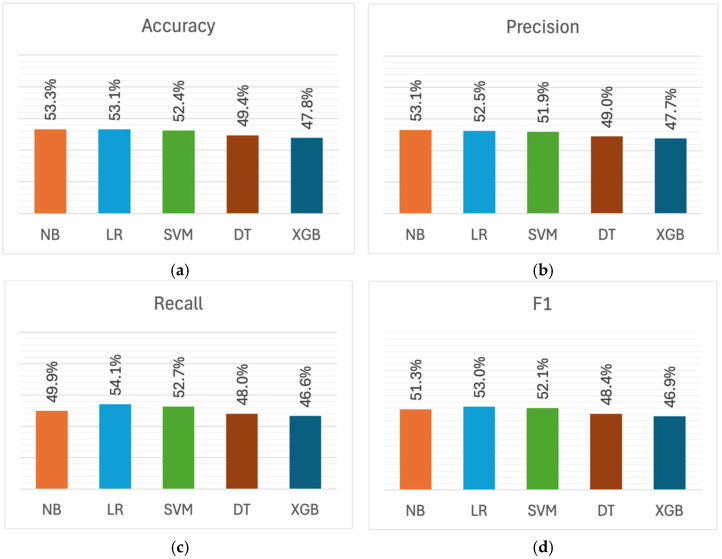
Performance metrics for cynicism detection across machine learning models: (**a**) Accuracy; (**b**) Precision; (**c**) Recall; (**d**) F1 score.

Figure 3 shows the performance metrics obtained by models in detecting cynicism. As illustrated in Figure 3a, NB, LR, and SVM demonstrated close accuracy (52.4–53.3%), with NB achieving the highest score of 53.3%. Conversely, the DT and XGBoost models obtained lower accuracies of 49.4% and 47.8%, respectively. The precision metrics displayed a similar pattern in Figure 3b, with NB achieving the highest at 53.1%, followed closely by LR (52.5%) and SVM (51.9%). DT and XGBoost achieved lower precision scores of 49% and 47.7%, respectively.

As depicted in Figure 3c, the recall metrics exhibited variation similar to accuracy and precision, with values ranging from 46.6% (XGBoost) to 54.1% (LR). This pattern is also reflected in the F1 scores shown in Figure 3d, where XGBoost recorded the lowest F1 score at 46.9%, while LR demonstrated higher performance (53.0%) compared to other models.

Unlike exhaustion detection, the models displayed relatively balanced precision and recall metrics in cynicism detection, with a precision-recall gap of about 1–3%. The largest gap was observed in NB (3.2 percentage points), which obtained higher precision (53.1%) than recall (49.9%). This indicates that NB tends to be more conservative in identifying cynicism cases, missing some actual cases rather than over-identifying them, unlike the exhaustion detection models, which had substantially higher recall than precision.

In general, NB and LR exhibited marginally superior performance in cynicism detection compared to other models, with NB obtaining the highest scores in accuracy and precision, while LR achieved the highest recall and F1 scores. DT and XGBoost performed below 50% in all metrics, with XGBoost consistently recording the lowest scores. Moreover, models were less effective in detecting cynicism compared to exhaustion. The mean F1 score dropped from 64.1% for exhaustion detection to 50.3% for cynicism detection—an average decline of 13.8 percentage points.

### 4.3. Professional Efficacy Detection Performance

**Figure 4:**
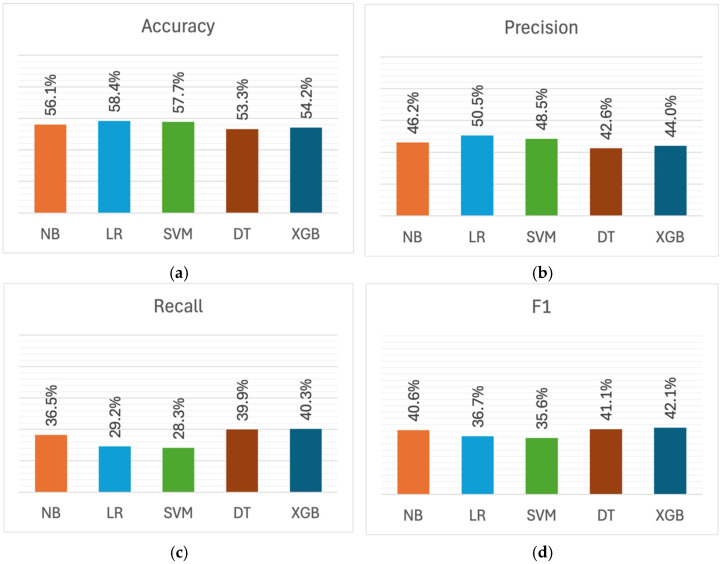
Performance metrics for professional efficacy detection across machine learning models: (**a**) Accuracy; (**b**) Precision; (**c**) Recall; (**d**) F1 score.

Figure 4 shows the performance metrics for professional efficacy detection models. Similar to the exhaustion dimension, models achieved similar accuracy (53.3–58.4%) in detecting professional efficacy, as can be seen in Figure 4a. LR emerged with the highest accuracy of 58.4%, followed by SVM with 57.7%. The lowest accuracy was obtained by DT at 53.3%.

As shown in Figure 4b, LR achieved the highest precision at 50.5%, whereas DT scored the lowest (42.6%). In the recall metric presented in Figure 4c, the highest score was attained by XGBoost at 40.3%, while the SVM model recorded the lowest at 28.3%. A similar observation can be made about F1 scores in Figure 4d: XGBoost obtained the highest F1 score of 42.1%, while the lowest F1 was achieved by SVM at 35.6%.

There was a notable disparity between precision and recall metrics. However, in contrast to exhaustion detection, these models consistently demonstrated higher precision than recall when detecting low professional efficacy. This pattern was particularly pronounced in LR and SVM, where LR obtained 50.5% precision and 29.2% recall, while SVM achieved 48.5% precision and 28.3% recall, creating a precision-recall gap of about 20 percentage points. This suggests that these models produced more false negatives, indicating that they tend to be more conservative in identifying students with low professional efficacy, missing actual cases rather than over-identifying them.

In general, the mean F1 score across all models was 39.2%, indicating that low professional efficacy was the most challenging dimension to detect, compared to exhaustion (64.1% mean F1) and cynicism (50.3% mean F1)

### 4.4. Feature Associations with Exhaustion

To get a better understanding of the classification performance, we followed up our evaluations with a closer investigation of features. As the majority of our input features were categorical (16 out of 18), we employed the chi-square (*χ*^2^) test of independence to examine the statistical relationship between each predictor and the specific burnout target variable. Given the exploratory nature of this analysis, we reported both *p*-values and effect sizes (Cramér’s V) to provide information about the magnitude and practical significance of associations. Additionally, we conducted feature selection using the Boruta algorithm to identify features contributing to model predictions and validate the statistical associations.

Table 4 shows the chi-square analysis results of the relationships between each feature and the exhaustion (EX) variable. The analysis revealed a significant association (*p* < 0.05) only with *Career* (*χ*^2^(2) = 6.843, *p* = 0.033, Cramer’s *V* = 0.110, small effect). Additionally, *Academic Level* (*χ*^2^(5) = 7.772, *p* = 0.169, Cramer’s *V* = 0.117), *Program* (*χ*^2^(3) = 7.408, *p* = 0.060, Cramer’s *V* = 0.115), and *Class of Honors* (*χ*^2^(7) = 5.998, *p* = 0.540, Cramer’s *V* = 0.103) demonstrated small effect sizes, but these associations were not statistically significant. The remaining features showed negligible effects.

To analyze the relationship between continuous features (i.e., *Cumulative GPA* and *Total Credit Hours*) and exhaustion, we conducted Mann-Whitney *U* tests. This non-parametric test was chosen due to the non-normal distribution of the variables. We examined differences between students experiencing high exhaustion and those who were not. As shown in Table 5, neither *Cumulative GPA* (*U* = 39,876.5, *p* = 0.664) nor *Total Credit Hours* (*U* = 38,810.5, *p* = 0.904) showed statistically significant differences between two groups.

### 4.5. Feature Associations with Cynicism

Table 6 shows the chi-square analysis results of the relationships between features and the cynicism (CY) variable. Statistically significant associations were observed only for *Gender* (*χ*^2^(1) = 5.203, *p* = 0.023, Cramer’s *V* = 0.096, negligible effect) and *Academic Status* (*χ*^2^(2) = 8.474, *p* = 0.014, Cramer’s *V* = 0.122, small effect). *Gender* showed a negligible effect size despite the statistical significance. *Academic Level* (*χ*^2^(5) = 5.952, *p* = 0.311, Cramer’s *V* = 0.103) and *Class of Honors* (*χ*^2^(7) = 8.261, *p* = 0.310, Cramer’s *V* = 0.121) demonstrated small effect sizes, although these associations were not statistically significant. The remaining features had negligible effect sizes.

Table 7 presents the results of Mann–Whitney *U* tests examining differences between students exhibiting high cynicism and those who were not. The analysis revealed a statistically significant difference in *Cumulative GPA* scores (*U* = 44,046, *p* = 0.033). Students exhibiting high cynicism (*Mdn* = 3.38, *IQR* = 0.78) had lower median *Cumulative GPA* scores compared to their peers not exhibiting cynicism (*Mdn* = 3.47, *IQR* = 0.71). In *Total Credit Hours* (*U* = 37,232.5, *p* = 0.168), no significant differences were found between the two groups.

### 4.6. Feature Associations with Professional Efficacy

Table 8 presents the chi-square analysis of the relationships between each feature and the professional efficacy (PE). Two features demonstrated significant association with PE, both with small effect sizes: *Nationality* (*χ*^2^(1) = 6.607, *p* = 0.010, Cramer’s *V* = 0.108, small effect) and *Race* (*χ*^2^(4) = 10.232, *p* = 0.037, Cramer’s *V* = 0.135, small effect). As with cynicism, *Class of Honors* (*χ*^2^(7) = 12.791, *p* = 0.077, Cramer’s *V* = 0.150) demonstrated a small effect size, but the association was not statistically significant.

Table 9 presents the results of Mann–Whitney *U* tests examining differences in *Cumulative GPA* and *Total Credit Hours* between students exhibiting low professional efficacy and those not exhibiting low professional efficacy. A statistically significant difference was found in *Cumulative GPA* scores (*U* = 44,073, *p* = 0.005). Students exhibiting low professional efficacy (*Mdn* = 3.33, *IQR* = 0.76) had lower median *Cumulative GPA* scores compared to students who reported higher professional efficacy (*Mdn* = 3.51, *IQR* = 0.78). In *Total Credit Hours* (*U* = 36,948.5, *p* = 0.364), no significant differences were observed between two groups.

## 5. Discussion

In this study, we investigated the feasibility of detecting academic burnout from university records using machine learning. We developed models to detect the presence of three major symptoms of burnout: exhaustion, cynicism, and low professional efficacy. The evaluation results demonstrated considerable variability between burnout dimensions, with models being more effective at detecting exhaustion (mean F1 score of 64.1%). The results suggest that student records used for burnout detection are better at capturing exhaustion than other burnout symptoms.

### 5.1. Detecting Exhaustion

The highest performance in this study was achieved by LR when predicting exhaustion, with an F1 of 68.4%. This finding is similar to the performance achieved by ref. [31]. Their best-performing models obtained an F1 score of 70.3% in detecting burnout among university students. However, an important distinction is that their models utilized features obtained from behavioral surveys (i.e., self-learning and social support questionnaires), whereas our method relied entirely on university records.

Our models showed notable differences when detecting exhaustion, with clear distinctions between model types. LR and SVM achieved similar performance, as both are linear classifiers that learn similar decision boundaries, albeit through different approaches. Moreover, they both emerged as the best-performing models, as can be seen from their recall and F1 scores.

DT showed lower performance in comparison to linear models. This performance gap potentially indicates that the data contained noise (e.g., irrelevant features), as decision trees are sensitive to noisy data [51]. This issue can be mitigated by combining multiple decision trees into an ensemble [52], as demonstrated with XGBoost, which obtained higher recall and F1 scores than a single DT model.

The higher performance of LR and SVM compared to tree models also potentially suggests that subtle indicators of exhaustion are distributed across multiple features. The rule-based DT models may struggle to capture these dispersed indicators. At each node, they create decision rules using individual features, which need to show sufficient distinctions between exhausted and non-exhausted students. On the other hand, linear models are particularly effective at integrating weak signals from multiple features [53].

Models tend to produce higher recall than precision in exhaustion detection, as observed in LR and SVM. This tendency suggests that these models are likely to overclassify exhaustion cases, categorizing students as exhausted when they are not, rather than missing actual cases. The class distribution (57% negative, 43% positive) could also contribute to this pattern. More negative (non-exhaustion) examples, combined with models’ tendency to over-identify exhaustion cases, inherently result in more false alarms, creating the pattern of higher recall and lower precision. While the exhaustion detection models produce false positives, the higher recall has important practical implications. High recall means the models miss few actual exhaustion cases, enabling early identification of at-risk students and allowing for a more proactive outreach.

### 5.2. Detecting Cynicism

Cynicism detection proved more challenging than exhaustion detection, which resulted in the mean F1 score declining by 13.8 percentage points, from 64.1% (EX) to 50.3% (CY). This gap suggests that cynicism is reflected less clearly in administrative records than exhaustion. Since cynicism represents a more internal psychological state, it can be more difficult to capture in university records.

Consistent with exhaustion detection, rule-based models (DT and XGBoost) showed lower performance than linear models. However, in this case, XGBoost performed worse than the simpler DT model across all metrics. LR and SVM emerged with stronger performance, particularly in recall and F1 scores. They were only exceeded by NB in terms of accuracy and precision, although by a modest margin. Similar to exhaustion, better performance of linear models could indicate that weak cynicism signals may be distributed across multiple features that rule-based partitioning struggles to delineate.

In contrast to exhaustion and professional efficacy, models obtained more balanced precision and recall scores when detecting cynicism. This likely stems from a balanced class distribution, as 50.27% of the examples belong to the “no cynicism” class. This reduces the inclination toward the higher recall observed in the imbalanced exhaustion dataset.

### 5.3. Detecting Professional Efficacy

Detecting low professional efficacy was particularly difficult, with models obtaining an average 39.2% F1 score, substantially lower than exhaustion (average F1 of 64.1%) and cynicism (average F1 of 50.3%). This represents a performance reduction of nearly 25 percentage points compared to exhaustion detection, suggesting that professional efficacy had the weakest representation in university records.

The poor PE detection rates could indicate that PE represents overall burnout very distinctly compared to EX and CY. The distinct nature of professional efficacy is often a major discussion point in burnout literature. Some researchers suggest that burnout is primarily a two-dimensional construct (EX and CY), omitting professional efficacy from their operational definitions [7]. For example, ref. [29] excluded the personal accomplishment dimension (equivalent of PE in the medical version of the MBI) when constructing their target variable for detecting burnout among healthcare professionals. Instead, they relied solely on exhaustion and depersonalization (CY equivalent in the medical MBI). Ref. [24] took a different approach to target variable creation for their teacher burnout detection models, and used the Oldenburg Burnout Inventory, which does not include a professional efficacy dimension.

Contrary to exhaustion and cynicism, tree-based models (DT and XGBoost) outperformed linear models (LR and SVM) in terms of recall and F1, when detecting low professional efficacy. Given the low performance levels, these shifts between model types may be more indicative of particularly weak professional efficacy signals in the data rather than meaningful differences between algorithms.

When it comes to professional efficacy detection, models tend to have higher precision than recall, despite having a similar class distribution to exhaustion detection (60% negative class). This pattern indicates that PE detection models are more conservative in classifying students as exhibiting low professional efficacy, producing a higher number of false negatives. This reversed pattern may be attributed to inverted class labels in the PE target variable. Specifically, in EX and CY prediction models, the positive class comprises cases with high exhaustion and cynicism, respectively. In PE detection models, the positive class consists of examples with low professional efficacy. This labeling convention reflects the Maslach burnout model, which explicitly defines burnout as the expressions of high exhaustion, high cynicism, and low professional efficacy.

### 5.4. Features Associated with Burnout Dimensions

Our approach to feature engineering is similar to refs. [17,36,37], although they focused on healthcare professionals. These studies utilized only existing hospital records to detect burnout, emphasizing the value of using passively collected data for developing burnout screening tools. However, these studies concluded that existing records had a limited ability to detect burnout. For example, ref. [36] reported modest predictive capability (AUC = 0.595) and found that none of the features derived from existing hospital records were able to identify clinicians with burnout.

Similarly, the features engineered from university records had limited discriminative ability for detecting student burnout. This was further supported by the statistical analysis of our features: out of 18 features, at most three of them had significant associations with the specific target variables. Only one feature *Career* had a statistically significant association with exhaustion. *Career* represents an academic program level and is an ordinal variable with three categories: 1 = “Foundation”; 2 = “Diploma”; and 3 = “Undergraduate.” This association suggests that students at different academic levels may experience varying degrees of exhaustion. However, the small effect size indicates that this relationship is relatively weak. Also, although not statistically significant, the *Program* feature’s *p*-value (*p* = 0.06) was the closest to the significance threshold (*p* < 0.05) among the remaining features. *Program* is a nominal variable representing specific academic programs: “B.C.S (Hons)”, “B.I.T. (Hons)”, “Dip. I.T.”, and “Foundation.” The relative salience of *Program*, combined with *Career’s* significant association, suggest that program-related factors may be among relevant indicators of exhaustion, possibly due to the different demands and expectations associated with different levels of education. This finding aligns with ref. [54], who similarly found significant differences in burnout among students at different academic levels.

Cynicism demonstrated associations with *Gender*, *Academic Status*, and *Cumulative GPA*. Although the association with *Gender* was statistically significant, its effect size was negligible, highlighting the weakness of this relationship. Previous research on gender differences in burnout has yielded mixed results. While some studies have identified significant gender-related variations in overall burnout [55,56,57], others have found no meaningful associations [10,25,58]. Refs. [2,12,59] reported gender associations with exhaustion and professional efficacy, but not with cynicism. Our findings uncovered an opposite pattern, with gender showing a significant association with cynicism but not with other two dimensions, suggesting potential variability in how burnout dimensions are expressed in different populations or contexts. Cynicism also showed significant associations with *Academic Status* and *Cumulative GPA*. These two features are closely related: *Academic Status* represents students’ current academic standing (“Pass”, “Probation”, or “Terminated-Reinstated”) and is determined based on the *Cumulative GPA* performance. The students exhibiting high cynicism had lower median *Cumulative GPA* scores (3.38) compared to those without cynicism (3.47). These associations suggest that students experiencing academic difficulties may develop cynicism toward their academic work. Being on probation and being previously terminated may also exacerbate cynical attitudes. These findings align with ref. [60], who found that lower GPA and previously failing a course were significantly associated with higher cynicism levels. However, it is important to note that this relationship could be bidirectional: poor academic performance may foster cynicism, while cynical attitudes could also undermine academic effort and performance.

Professional efficacy demonstrated significant associations with three features: *Nationality, Race*, and *Cumulative GPA*. *Nationality* (coded as local vs. international students) and *Race* both had small effect sizes, suggesting potential differences in how students from different backgrounds perceive their academic competence, though the underlying mechanisms require further investigation. The *Cumulative GPA* association was particularly notable, with students exhibiting low professional efficacy showing lower median *Cumulative GPA* scores (3.33) compared to those with higher professional efficacy (3.51). This pattern was expected, given that professional efficacy measures students’ perceived academic competence. Our finding is consistent with refs. [56,60], who similarly found positive associations between GPA and professional efficacy.

The importance of *Cumulative GPA* for cynicism and professional efficacy was further supported by machine learning-based feature selection technique. Having applied Boruta algorithm to identify important features, we observed similar trends as discovered in our statistical analysis. Specifically, for cynicism and professional efficacy, Boruta selected only *Cumulative GPA* as an important feature. This aligns with the Mann–Whitney *U* test results showing significant *Cumulative GPA* differences between burnout and non-burnout groups for these two dimensions. In contrast, Boruta selected no features as important for exhaustion detection, consistent with the limited statistical associations and negligible to small effect sizes observed in chi-square tests.

### 5.5. Limitations and Future Directions

The primary limitation of this study stems from the modest performance, particularly for professional efficacy. These results underscore the challenges in capturing psychological constructs through administrative data alone. In particular, the university records lack behavioral indicators. Burnout is primarily a psychological syndrome that manifests as behavioral, psychological, and physiological changes. The behavioral patterns collected with surveys have a greater discriminative capacity in detecting burnout. For example, ref. [24] uncovered that survey-based features that capture problematic internet use (i.e., internet addiction) were among the best predictors of burnout among teachers. Ref. [34] showed that adding survey-collected behavioral features (caffeine intake, alcohol consumption) improved burnout detection performance from a 67% F1 score to 81%, compared to using sensor data alone.

Given this limitation, future studies could incorporate records about students’ involvement in university societies and clubs. This information could serve as behavioral indicators of student engagement and social connection. They could improve model performance since extracurricular activities have been shown to be correlated with burnout [55,60]. In addition, future works could explore additional variables that are better at capturing burnout. Currently, our dataset consists of 18 features, the majority of which are categorical. Incorporating sociodemographic variables—such as age, marital status, and household size—could enhance detection as they have established correlations with burnout [2,37]. Such variables are typically collected by the university administration upon enrollment of the student, making it readily accessible for research purposes. Additionally, future work could incorporate temporal features such as CGPA history and attendance records. These features could capture important behavioral and academic trends, potentially revealing declining engagement and performance that may signal developing burnout.

## 6. Conclusions

This study investigated whether machine learning models could detect academic burnout symptoms using university records. Our results suggest that, while automated detection is feasible, the effectiveness varies considerably across different burnout dimensions. Models performed best at detecting exhaustion (with LR obtaining the highest F1 of 68.4%) but struggled with professional efficacy detection. Although university records have restricted discriminative ability in detecting burnout among students, as further supported by the limited statistically significant associations between our features and burnout dimensions, the findings of this study provide a foundation for future research in unobtrusive and automated student burnout detection, highlighting both the potential and challenges of using passively collected university records.

## Figures and Tables

**Table 1:** Class distribution across burnout dimensions.

Dimension	Class	
	No Burnout (Coded: 0)	Burnout (Coded: 1)
Exhaustion	324 (57.35%)	241 (42.65%)
Cynicism	284 (50.27%)	281 (49.73%)
Professional Efficacy	332 (58.76%)	233 (41.24%)

**Table 2:** Feature summary.

No.	Feature	Type	Description	Possible Values
1	Career	Ordinal	Level of academic program enrollment	1 = Foundation; 2 = Diploma; 3 = Undergraduate
2	Program Status	Binary	Current program completion status	Completed Program (0); Active in Program (1)
3	Current Term	Binary	Length of the current trimester	Short (0); Long (1)
4	Academic Level	Ordinal	Current year level within program type	1 = First year foundation; 2 = First year diploma; 3 = Second year diploma; 4 = First year bachelor’s; 5 = Second year bachelor’s; 6 = Third year bachelor’s
5	Campus	Binary	Campus location	Cyberjaya (0); Malacca (1)
6	Program	Nominal	Specific academic program enrolled	B.C.S (Hons); B.I.T. (Hons); Dip. I.T.; Foundation
7	Faculty	Binary	Faculty of enrollment	Faculty of Computing & Informatics (0); Faculty of Information Science & Technology (1)
8	Nationality	Binary	Student nationality status	International (0); Local (1)
9	Race	Nominal	Ethnic group classification	Chinese; Indian; Malay; Others (Malaysian); Others (non-Malaysian)
10	Gender	Binary	Student’s gender	Female (0); Male (1)
11	Discount	Binary	Status indicating if a student receives a tuition discount	No (0); Yes (1)
12	MUET Score	Binary	Status indicating if the student has a Malaysian University English Test score	No (0); Yes (1)
13	Financial Assistance	Binary	Status indicating if a student receives any form of financial assistance	No (0); Yes (1)
14	Loan	Binary	Status indicating if a student receives an educational loan	No (0); Yes (1)
15	Cumulative GPA	Continuous	Student’s cumulative grade point average	Theoretical range: 0.00–4.00; Range in the dataset: 1.14–4.00
16	Academic Status	Nominal	Student’s current academic standing	Pass; Probation; Terminated-Reinstated
17	Class of Honors	Nominal	Academic achievement classification	Credit; Distinction; First Class; Less 2; Pass; Second Class (Upper); Second Class (Lower); Third Class
18	Total Credit Hours	Continuous	Total academic credits earned	Range in dataset: 5–116

**Table 3:** Model hyperparameters.

Model	Python 3.9 Package	Key Hyperparameters
NB	sklearn.naive_bayes.BernoulliNB	alpha = 1.0 (smoothing parameter)
LR	sklearn.linear_model.LogisticRegression	C = 1.0 (regularization), penalty = ‘l2’, solver = ‘lbfgs’, max_iter = 1000, random_state = 42
SVM	sklearn.svm.LinearSVC	C = 1.0 (regularization), penalty = ‘l2′, loss = ‘squared_hinge’, max_iter = 1000, random_state = 42
DT	sklearn.tree.DecisionTreeClassifier	criterion = ‘gini’, max_depth = None, min_samples_split = 2, min_samples_leaf = 1, random_state = 42
XGBoost	xgboost.XGBClassifier	n_estimators = 100, learning_rate = 0.3 (default), max_depth = 6 (default), objective = ‘binary:logistic’, random_state = 42

**Table 4:** Chi-square test results examining associations between features and exhaustion dimension.

Feature	*χ* ^2^	*df*	*p*-Value	Significant	Cramer’s *V*	Effect Size
Career	6.843	2	0.033	Yes	0.110	Small
Program Status	0.000	1	1.000	No	0.000	Negligible
Current Term	1.625	1	0.202	No	0.054	Negligible
Academic Level	7.772	5	0.169	No	0.117	Small
Campus	0.013	1	0.908	No	0.005	Negligible
Program	7.408	3	0.060	No	0.115	Small
Faculty	0.013	1	0.908	No	0.005	Negligible
Nationality	0.989	1	0.320	No	0.042	Negligible
Race	1.379	4	0.848	No	0.049	Negligible
Gender	0.797	1	0.372	No	0.038	Negligible
Discount	0.000	1	0.999	No	0.000	Negligible
MUET Score	0.228	1	0.633	No	0.020	Negligible
Financial Assistance	0.525	1	0.469	No	0.030	Negligible
Loan	0.018	1	0.893	No	0.006	Negligible
Academic Status	4.160	2	0.125	No	0.086	Negligible
Class of Honors	5.998	7	0.540	No	0.103	Small

**Table 5:** Mann–Whitney *U* test results comparing students with and without exhaustion.

Feature	*Mdn* (No EX)	*Mdn* (EX)	*IQR* (No EX)	*IQR* (EX)	*U*-Statistic	*p*-Value	Significant
Cumulative GPA	3.46	3.41	0.76	0.76	39,876.5	0.664	No
Total Credit Hours	44.00	46.00	53.00	47.00	38,810.5	0.904	No

**Table 6:** Chi-square test results examining associations between features and cynicism dimension.

Feature	*χ* ^2^	*df*	*p*-Value	Significant	Cramer’s *V*	Effect Size
Career	3.291	2	0.193	No	0.076	Negligible
Program Status	0.000	1	1.000	No	0.000	Negligible
Current Term	0.287	1	0.592	No	0.023	Negligible
Academic Level	5.952	5	0.311	No	0.103	Small
Campus	0.297	1	0.586	No	0.023	Negligible
Program	4.438	3	0.218	No	0.089	Negligible
Faculty	0.297	1	0.586	No	0.023	Negligible
Nationality	0.000	1	1.000	No	0.000	Negligible
Race	0.558	4	0.968	No	0.031	Negligible
Gender	5.203	1	0.023	Yes	0.096	Negligible
Discount	0.072	1	0.788	No	0.011	Negligible
MUET Score	0.323	1	0.570	No	0.024	Negligible
Financial Assistance	0.801	1	0.371	No	0.038	Negligible
Loan	0.151	1	0.698	No	0.016	Negligible
Academic Status	8.474	2	0.014	Yes	0.122	Small
Class of Honors	8.261	7	0.310	No	0.121	Small

**Table 7:** Mann–Whitney *U* test results comparing students with and without cynicism.

Feature	*Mdn* (No CY)	*Mdn* (CY)	*IQR* (No CY)	*IQR* (CY)	*U*-Statistic	*p*-Value	Significant
Cumulative GPA	3.47	3.38	0.71	0.78	44,046.0	0.033	Yes
Total Credit Hours	44.00	46.00	56.25	45.00	37,232.5	0.168	No

**Table 8:** Chi-square test results examining associations between features and professional efficacy dimension.

Feature	*χ* ^2^	*df*	*p*-Value	Significant	Cramer’s *V*	Effect Size
Career	1.957	2	0.376	No	0.059	Negligible
Program Status	0.000	1	1.000	No	0.000	Negligible
Current Term	0.518	1	0.472	No	0.030	Negligible
Academic Level	5.329	5	0.377	No	0.097	Negligible
Campus	0.424	1	0.515	No	0.027	Negligible
Program	2.539	3	0.468	No	0.067	Negligible
Faculty	0.424	1	0.515	No	0.027	Negligible
Nationality	6.607	1	0.010	Yes	0.108	Small
Race	10.232	4	0.037	Yes	0.135	Small
Gender	0.000	1	1.000	No	0.000	Negligible
Discount	0.324	1	0.569	No	0.024	Negligible
Muet Score	0.001	1	0.980	No	0.001	Negligible
Financial Assistance	0.422	1	0.516	No	0.027	Negligible
Loan	0.264	1	0.607	No	0.022	Negligible
Academic Status	4.822	2	0.090	No	0.092	Negligible
Class of Honors	12.791	7	0.077	No	0.150	Small

**Table 9:** Mann–Whitney *U* test results comparing students with and without low professional efficacy.

Feature	*Mdn* (Not Low PE)	*Mdn* (Low PE)	*IQR* (Not Low PE)	*IQR* (Low PE)	*U*-Statistic	*p*-Value	Significant
Cumulative GPA	3.51	3.33	0.78	0.76	44,073.0	0.005	Yes
Total Credit Hours	44.50	46.00	52.00	47.00	36,948.5	0.364	No

## Data Availability

The data presented in this study are available on request from the corresponding author. The data are not publicly available due to is not readily available because of data privacy and restrictions from the funding agency.

## Abbreviations

The following abbreviations are used in this manuscript:

AUC	Area Under the Curve
CS	Computer Science
CY	Cynicism
DT	Decision Tree
ECG	Electrocardiogram
EHR	Electronic Health Record
EX	Exhaustion
FN	False Negative
FP	False Positive
GBDT	Gradient Boosting Decision Tree
GPA	Grade Point Average
IT	Information Technology
LR	Logistic Regression
MBI	Maslach Burnout Inventory
MBI-GS(S)	Maslach Burnout Inventory-General Survey for Students
MLP	Multilayer Perceptron
MUET	Malaysian University English Test
NB	Naive Bayes
PE	Professional Efficacy
RF	Random Forest
SVM	Support Vector Machine
TN	True Negative
TP	True Positive
XGBoost	Extreme Gradient Boosting
